# Digital platform for planning facial asymmetry orthodontic-surgical treatment preparation

**DOI:** 10.1590/2177-6709.23.3.080-093.sar

**Published:** 2018

**Authors:** Octavio Cintra, Simonas Grybauskas, Carlos Jorge Vogel, Dalia Latkauskiene, Nilo Alves Gama

**Affiliations:** 1 Private practice (São Paulo/SP, Brazil).; 2 Private practice (Vilnius, Lithuania).; 3 Vilnius University Hospital Zalgirio Clinic, Department of Oral of Maxillofacial Surgery (Vilnius, Lithuania).; 4 Lithuanian University of Health Sciences, Department of Plastic and Reconstructive Surgery (Kaunas, Lithuania).; 5 Universidade Federal da Bahia (Salvador/BA, Brazil).; 6 Private practice (Barueri/SP, Brazil).

**Keywords:** Facial asymmetry, Virtual planning, Mandibular hyperplasia

## Abstract

Dentofacial deformities usually are surgically treated, and 3D virtual planning has been used to favor accurate outcomes. Cases reported in the present article show that orthognathic surgery carried out to correct facial asymmetries does not comprise only one treatment protocol. 3D virtual planning might be used for surgical planning, but it should also be used to diagnose the deformity, thus allowing for an analysis of the best-recommended possibilities for the orthodontic preparation that suits each individual case.

## RESUMO

As deformidades dentofaciais são, geralmente, tratadas de forma cirúrgica, e o planejamento virtual 3D tem sido utilizado para aumentar a precisão dos resultados. Os casos exemplificados no presente artigo mostram que a cirurgia ortognática para correção das assimetrias faciais não apresenta um único protocolo de tratamento. O planejamento virtual 3D pode ser adotado para planejar a cirurgia, mas também deve ser utilizado na fase de diagnóstico da deformidade, assim permitindo uma análise das possibilidades mais indicadas para o preparo ortodôntico mais adequado em cada caso.

## INTRODUCTION

Facial growth must occur with balance, in which bone structures depend on each other for correct facial growth and position. Whenever balance is interrupted by external factors or inherent factors, the individual might develop facial asymmetry.

Facial asymmetry etiology, whether genetic, pathological, functional or traumatic, is important to guide treatment.

Due to biological factors inherent to the development processes as well as to environmental disturbances, perfect bilateral symmetry is rarely found.^1^

Orthodontic literature usually addresses changes in anteroposterior and vertical planes. However, little attention is given to craniofacial changes in frontal view.^2^ ^,^ ^3^

Orthognathic surgery is the treatment option for asymmetrical adult patients. Dentofacial anatomy is of a complex nature and, for this reason, orthognathic surgery often requires thorough planning.^4^ Even though surgical techniques have presented fast improvements in the last 50 years - such as rigid internal fixation, use of resorbable materials, and distraction osteogenesis -, tools available for orthognathic surgery planning have not undergone any changes since the 1960s, and neither have two-dimensional cephalometry (2D), prediction tracing, and cast surgery.^5,6^ A number of problems have been documented in association with the aforementioned conventional techniques, which have often have led to unsatisfactory outcomes.^6^

Virtual planning has arisen as an important tool for facial asymmetry planning, as it allows for greater accuracy in asymmetrical patients treatment, from both surgical and orthodontic perspectives.

Patients subjected to orthognathic surgery usually undergo a orthodontic preparatory step before the surgical procedure, which lasts on average for 12 to 18 months.^24-26^ 3D virtual platform is normally used at a further preparatory step of which aim is to have surgical movements planned.

Thus, the present article aims at demonstrating and discussing how 3D virtual planning not only aids surgical planning, but also allows decisions made during orthodontic preparation to be determined on the basis of anatomic diagnosis of the deformity and foreknowledge of movements necessary for orthognathic surgery.

## LITERATURE REVIEW

Clinical expression of facial asymmetry occurs when there is a bone deviation of at least 4mm. Asymmetries with values under 4mm are considered subclinical.^2,12^ Nevertheless, clinical expression of asymmetry or its disguise will depend on individual characteristics, such as soft tissue thickness in the region that is out of balance.^8^

As for classification of craniofacial asymmetries, Bishara et al^10^ assessed the structures involved and established that asymmetries could be classified as dental, skeletal, muscular or functional.

Dentofacial deformities might comprise isolated asymmetries of the maxilla or maxillomandibular asymmetries.^8^ As suggested by Obwegeser,^9^ asymmetries have been grouped as: Type I or hemimandibular hyperplasia, Type II or hemimandibular elongation, and Type III or hybrid (a combination of Types I and II).

Tridimensional increase in mandibular volume on the affected side, from condyle to symphysis, is typical of Type I. Nevertheless, the symphysis is not deviated to the contralateral side and crossbite is absent. Elongation of the mandible on the affected side is typical of Type II, which results in chin deviation to the contralateral side and crossbite; there is no increase in volume on the affected side. An increase in volume on the affected side combined with elongation of the mandible is typical of Type III, in addition to displacement of chin to the contralateral side; it is a hybrid form (a combination of Types I and II).^9^

In many patients, asymmetry results from a series of dentofacial changes, and might be associated with postural compensations that hinder the correct characterization of the disharmony.^13^ Clinicians need to determine which tissues are the cause of asymmetry and whether they have a primary or secondary involvement. That piece of information will, in turn, establish treatment planning. For instance, soft tissue facial asymmetry caused by mandibular asymmetry needs to be corrected by means of skeletal correction; whereas soft tissue deformity caused by hemifacial microssomia often requires more attention.^20^

Facial asymmetry must be assessed by thorough and judicious analysis conducted during patient’s first interview, extra- and intraoral clinical examination, as well as supplementary and diagnostic examination.^14^ ^,^ ^15^

During patient’s first interview, his/her complaints, expectations and history must be recorded, while investigating data related to potential infections, trauma or craniofacial pathologies.

Clinical examination allows for analysis and further clinical perception of asymmetry in sagittal, lateral and vertical planes. This is the most important diagnostic tool for facial asymmetry assessment.^10,16^ Facial assessment comprises visual inspection of facial morphology combined with soft and hard tissues as well as TMJ palpation.^13^ Complete facial assessment must be carried out, with special attention to the central portion of the chin, lip commissures leveling, bilateral symmetry of gonial angles, and symmetry of mandibular body contour.^13^

At smiling, analysis should assess whether dental midlines coincide with facial midline, inclination of the occlusal plane and the amount of bilateral gingival exposure. Intraoral clinical examination should assign considerable importance to assessment of occlusion, posterior and anterior teeth tipping, presence of crossbite and functional deviation of mandible.^11^ ^,^ ^16^ ^-^ ^18^

In order to have asymmetry assessed, patient must be in upright position, looking forward, with teeth in centric occlusion and relaxed lips. Patient’s upper and lower views often also help in establishing the degree of asymmetry.^13^ Midline is taken in centric relation (clinical) and at first tooth contact. Should condyles be out of centric position, it is impossible to have midline assessed.^35^ A possible procedure is to use a ruler or a wire stretched out from the glabella region to the region located below the chin, going over the central portion of the philtrum.^19^ ^,^ ^35^

Facial asymmetry correction planning can be simplified by clinical simulation. The mandible is subjected to rotation in the axial plane and a new starting point is determined for the planning procedure. The patient is required to rotate the mandible in order to move the chin towards the facial midline, causing the soft tissue of gonial angle to appear as symmetrical as possible. Whether chin deviation is corrected, under-corrected or overcorrected, simulation goal is to correct soft tissue symmetry in the posterior face. Forced symmetry position is recorded and serves as starting point for the entire planning procedure. Particular attention should be given to the strong possibility of change in philtrum position after this maneuver (forced symmetry protocol).^27^

Occlusal plane inclination is assessed especially in asymmetric patients. The patient is required to bite a wooden spatula. The occlusal plane is taken in relation to the interpupillary plane. However, for this assessment modality, it is necessary to ensure not only that orbits are in a horizontal plane, but also that orbital deformity is absent.^20^ Should deformity be present, orbits must be ignored and patient’s horizontal facial plane taken into consideration.

Clinical examination should be supplemented with other diagnostic tools, such as casts, photographs, radiographs, tomography and bone scintigraphy, in order to precisely locate and measure the structures involved in asymmetry.^11^ ^,^ ^21^

Studies have shown that single photon emission computed tomography (SPECT) is more sensitive when it comes to identifying active condylar hyperplasia, in comparison to bone scintigraphy used for probable clinical diagnosis of the same condition.^22^ ^,^ ^23^

In facial asymmetry treatment, soft tissue changes occurring after orthognathic surgery are not always ideal as expected,^28^ since soft tissue response is rather unpredictable. A number of studies on facial asymmetry do not include gonial angle soft tissue changes and take nose and chin soft tissue changes into account.^29-31^ A study conducted by Hagënsli et al^32^ found treatment outcomes were positively evaluated in more than 50% of patients, even in cases without complete asymmetry correction.

## TREATMENT

In both surgical or orthodontic treatment planning, strong emphasis should be given to diagnosis of asymmetry and patient’s final facial balance. Additionally, dental midlines must coincide, and there should be proper occlusion.^1^ ^,^ ^3^

Diagnosis of asymmetry can be easily achieved by the orthodontist in cases of significantly deviated dental midlines, in which there are no missing teeth, anomalies of shape or significant crowding on only one side of the dental arch.^3^ ^,^ ^10^ ^,^ ^33^

Asymmetry might be disguised by dental compensation. Should not be diagnosed, it tends to be revealed during orthodontic treatment, thus postponing treatment time and hindering its outcomes.^8^ Depending on the duration of the asymmetry and its severity, a considerable number of orthodontic and orthopedic options is described in the literature for facial asymmetry resolution.^8^ The following are among treatment approaches that have been proposed: asymmetrical mechanics, asymmetrical extractions or surgical interventions.^19^ ^,^ ^25^

**Figure 1:**
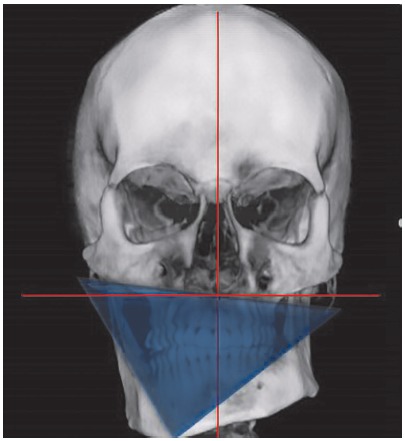
Prism used for tridimensional assessment of maxillomandibular complex symmetry.

The maxillomandibular complex is a tridimensional structure. To simplify tridimensional changes in skeletal and dental structures, as well as necessary soft tissue changes, a prism can be built to represent the maxillomandibular complex (Fig 1). This prism helps to see and assess different tridimensional surgical movements.^20^

**Figure 2:**
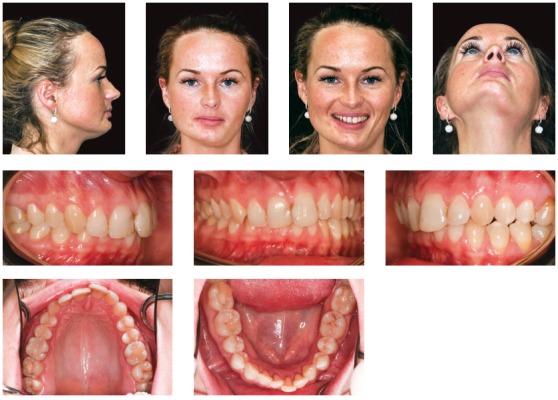
27-year-old patient with active condylar hyperplasia on the right side, inclination of the occlusal plane in both maxilla and mandible.

The use of 3D virtual platform for previous surgical planning helps choosing the best protocol. Cases with patients (Fig 2) presenting hemimandibular elongation might result in vertical asymmetry of gonial angles and inclination of the mandibular inferior border.^37^

**Figure 3:**
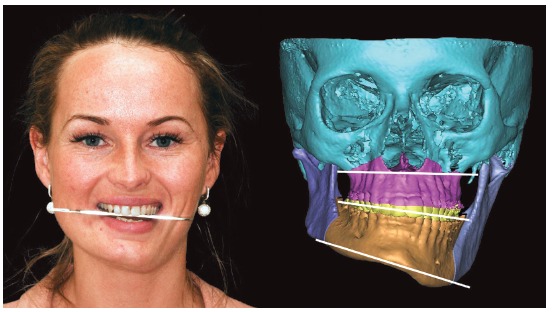
Relationship established between occlusal plane and maxillary and mandibular inferior borders.

As a consequence, we notice that due to an associated facial growth, compensatory changes usually occur in dentoalveolar processes. In those cases, there is inclination of the occlusal plane in the maxilla, aiming at compensating improper mandibular growth. As a result of compensatory action, inclination of the occlusal plane in frontal view is different from inclination of mandibular and maxillary inferior borders (Fig 3). 

In the following cases of facial asymmetry caused by hemimandibular elongation, two different recommended protocols are illustrated.

## CASE 1

Orthognathic surgery in asymmetrical patients combined with osteotomy of mandibular inferior border is a simple and stable procedure; however, in some cases, it requires inferior alveolar nerve (IAN) lateralization before bone resection.^34^

**Figure 4:**
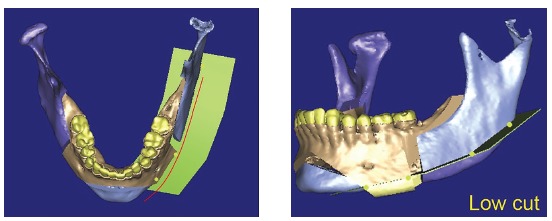
3D virtual planning simulating an osteotomy of the mandibular inferior border - low level.

**Figure 5:**
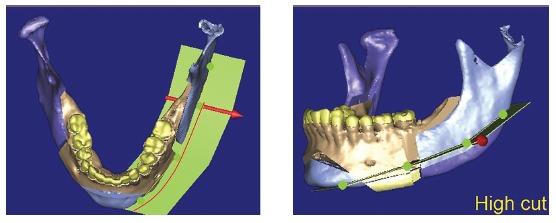
3D virtual planning simulating an osteotomy of mandibular inferior border - high level.

Another factor that should be assessed is osteotomy height for resection of the mandibular inferior border. The higher the cut line, the narrower the mandibular ramus remnant (Figs 4 and 5),^27^ which might result in remaining lateral asymmetry.

**Figure 6:**
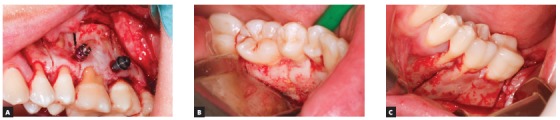
A) Corticotomies in the maxilla; B, C) Corticotomies in the region of mandibular molars and premolars lingually and buccally.

**Figure 7:**
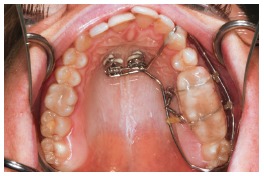
Skeletal anchorage achieved with two mini-implants and one mini-plate for intrusion.

**Figure 8:**
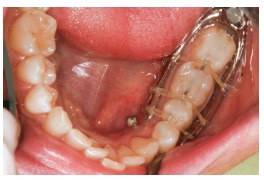
Skeletal anchorage achieved with one conventional implant in the retromolar region.

Thus, for this specific case, the decision was for maxillary and mandibular teeth intrusion on the hemimandibular elongation side. To enhance and speed up intrusion, corticotomies were carried out (Fig 6). For better anchorage and intrusion of teeth, mini-implants were placed in the median hard palate, whereas anchorage plates were placed in the maxilla (Fig 7). A conventional implant was placed in the retromolar region of the mandible (Fig 8), as an aid to anchorage and to increase efficiency of outcomes produced by the applied force.^37^

**Figure 9:**
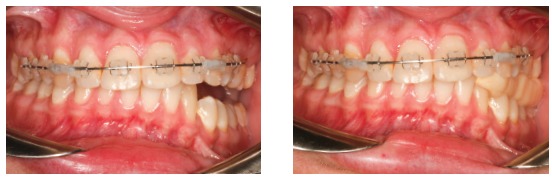
Intrusion of maxillary and mandibular teeth carried out four months after anchorage devices placement. A splint was gradually manufactured for occlusion retention on the left side.

**Figure 10:**
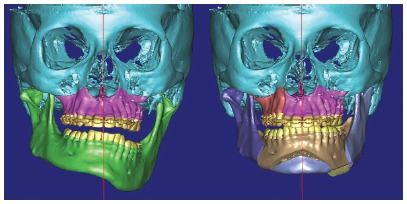
3D surgical virtual planning determining the amount of osteotomy of mandibular inferior border in 5mm.

Following steps were orthodontic decompensation and subsequent manufacture of a splint aimed at making posterior open bite stable on the left side (Fig 9).^37^ Virtual surgical simulation (SimPlant 13.0 software) was carried out, thus showing that the need for osteotomy of the mandibular inferior border had been reduced to 5mm (Fig 10).^37^

The surgical step was carried out as follows:

1. Le Fort I osteotomy with segmentation of maxilla between lateral incisor and canine on the right side.
2. Sagittal osteotomy of mandible on both sides.
3. Condylectomy on the left side.
4. 5-mm resection of mandibular inferior border on the left side.
5. Soft tissue suspension on the left side.
6. Genioplasty.

**Figure 11:**
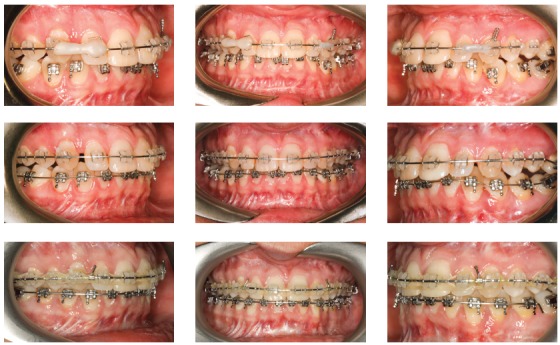
Occlusion development by means of postoperative orthodontic treatment.

**Figure 12:**
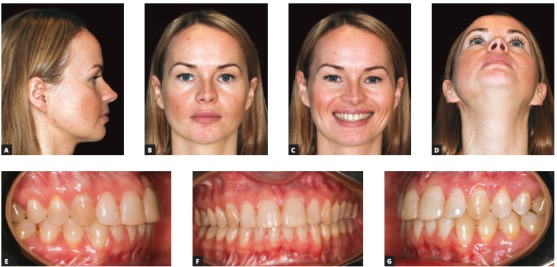
Final treatment after 1-year postoperative follow-up.

**Figure 13:**
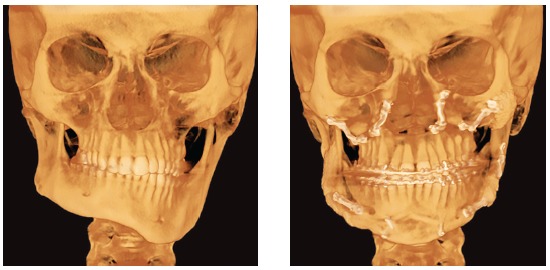
3D reconstruction before surgery and 10 months later.

**Figure 14:**
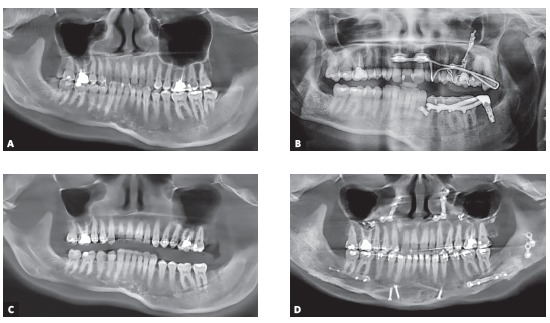
Panoramic radiographs: A) Before molar intrusion; B) During molar intrusion; C) Before orthognathic surgery; D) 10 months after orthognathic surgery.

At post-operative phase after three months, orthodontic treatment was resumed and included mandibular teeth alignment, closure of diastemata caused by segmentation of maxilla, and occlusion achievement (Fig 11). A 0.016 x 0.022-in NiTi wire was placed in the upper arch, whereas a 0.014-in NiTi wire was placed in the lower arch. Midline, sagittal discrepancies and vertical control were addressed with stainless steel 0.018 x 0.025-in wire in the upper arch and 0.018 x 0.025-in NiTi wire in the lower arch, followed by Class II elastics on the left side and subsequently followed by Class I elastics on both sides at treatment completion. Patient’s 1-year follow-up revealed stable occlusion and a resulting symmetrical facial frame (Fig 12). Imaging examination shows skeletal changes achieved before and after surgery (Figs 13 and 14).^37^

## CASE 2

**Figure 15:**
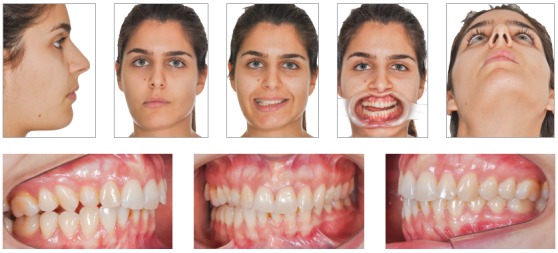
20-year-old patient, hemimandibular elongation on the right side with inclination of the occlusal plane in both maxilla and mandible.

The true vertical line (TVL)^35^ is important for profile clinical analysis; however, in asymmetrical patients, frontal analysis is more important to assess the facial frame when diagnosing the deformity (Fig 15).In the present case, imaging examination revealed the right mandibular condyle with changed morphology, when compared to the left one (Fig 17).

**Figure 16:**
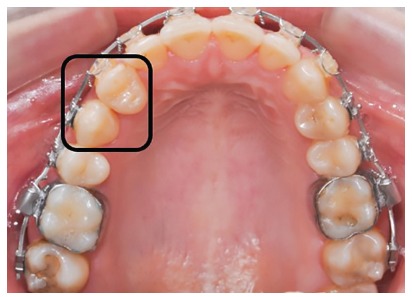
Tooth transposition between #13 and #14.

**Figure 17:**
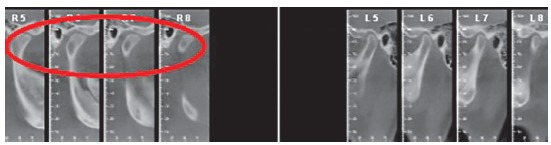
Computer tomography sagittal slice revealing changes in condylar morphology.

**Figure 18:**
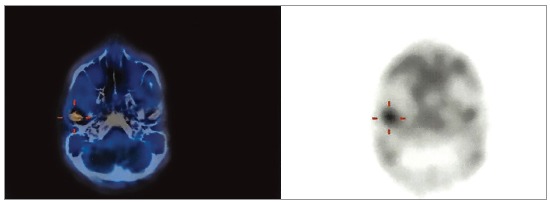
SPECT/CT with hypercaptation of mandibular condyle on the right side.

Single photon emission computed tomography (SPECT/CT) was requested and revealed hypercaptation of mandibular condyle on the right side (Fig 18). Imaging examination supplemented clinical facial analysis in the diagnostic hypothesis of Type II condylar hyperplasia, demanding a high condylectomy combined with orthognathic surgery.^36^

**Figure 19:**
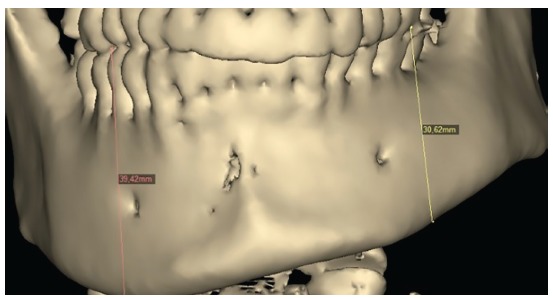
9-mm difference in height between mandibular body on right and left sides.

**Figure 20:**
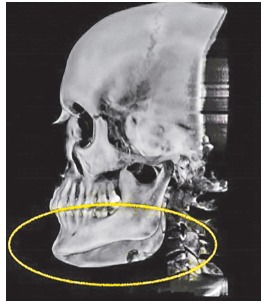
Computed tomography in lateral view evinces asymmetry between mandibular bodies.

**Figure 21:**
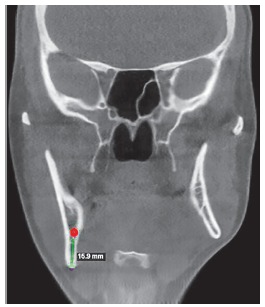
15.9-mm distance between the mandibular canal on the right side and the mandibular inferior border.

Difference in height between mandibular body on the right and left sides (Figs 19 and 20) was evinced, and so was the distance between the mandibular canal on the right side and its inferior border (Fig 21).

**Figure 22:**
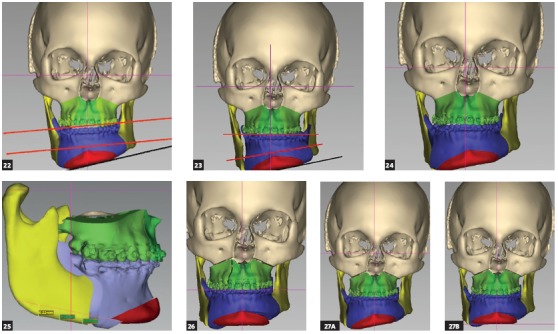
Difference in occlusal plane inclination in relation to the mandibular basal plane. Figure 23: Mandibular cant correction. Figure 24: Mandibular yaw correction. Figure 25: Required amount of osteotomy of mandibular inferior border. Figure 26: Maxilla segmentation planning: between canines and lateral incisors. Figure 27: Chin asymmetry correction.

Reverse planning allowed to assess the 9-mm difference between right and left mandibular bodies, in addition to the 15.9-mm distance between the mandibular canal and the mandibular inferior border. Based on the aforementioned measures, reverse surgical planning was carried out in the 3D virtual platform with the software NemoFAB (Nemotec S.L., Madrid, Spain). Both cant (inclination of the occlusion plane) and yaw (posterior rotation of arches) were corrected, as they are important movements for asymmetry correction (Figs 22-27). 

Surgical simulation allowed for the conclusion that mandibular cant correction (Fig 23) led to mandibular plane rotation, thus decreasing mandibular ramus height on the right side (mandibular segment in blue). After cant and yaw correction (Fig 24), we concluded the amount of mandibular resection needed was smaller than before (Fig 19), since there was a decrease in mandibular body height on the right side in the distal mandibular segment (in blue). Note a decrease of around 6mm in measures relative to the difference between right and left sides after correction (Fig 25). As a result, the need for orthodontic intrusion in the posterior region on the right side was eliminated. Thus, orthodontic procedures in preparation for surgery were naturally followed with alignment and leveling of arches with a rectangular wire sequence reaching stainless steel 0.019 x 0.025-in wire. Additionally, inferior alveolar nerve (IAN) lateralization was avoided (although it is often a feasible alternative). Due care must be given to assessment of skeletal asymmetry in frontal view while analyzing mandibular ramus remnant after osteotomy of the mandibular inferior border.^37^

**Figure 28:**
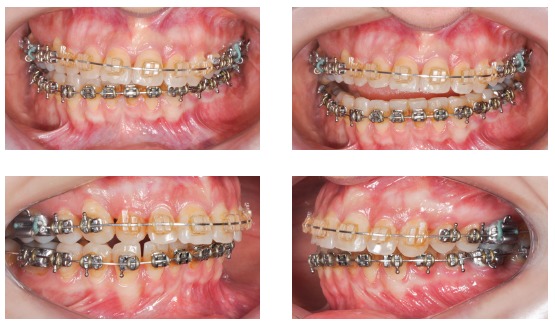
Orthodontic appliances with rectangular wires, hooks and molar bands for subsequent orthognathic surgery.

Subsequently, orthodontic decompensation was carried out. Restorative and periodontal treatment planning was carried out to disguise transposition of teeth #13 and #14 (Figs 16 and 28). 

At surgical step, the following was carried out:

1. Joint surgery for high condylectomy on the right side.
2. Sagittal osteotomy of mandible.
3. 6-mm resection of mandibular inferior border on the right side.
4. Autograft in mandibular body on the left side with mandibular inferior border fragment.
5. Le Fort I osteotomy with segmentation between lateral incisor and canine on both sides.
6. Genioplasty.

**Figure 29:**
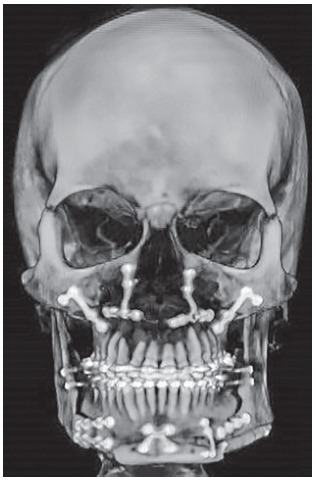
Tomographic scan four weeks after surgery.

**Figure 30:**
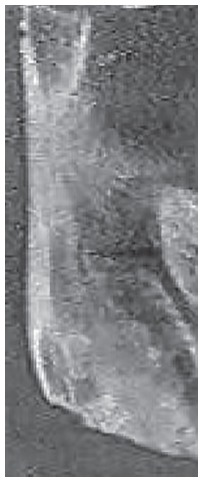
Tomographic scan revealing the right condyle where high condylectomy was carried out.

Using CT scan, it was possible to evaluate the four-week postoperative period (Figs 29 and 30).

**Figure 31:**
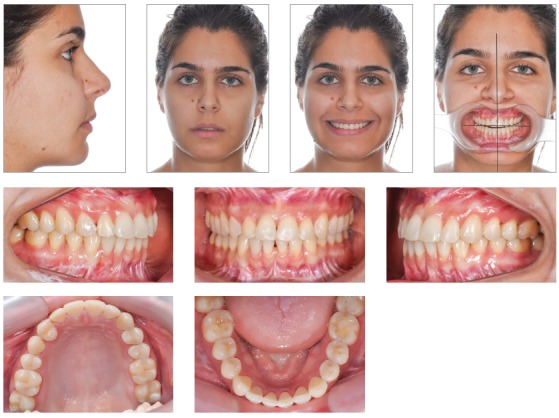
Final treatment, after 1-year postoperative follow-up.

**Figure 32:**
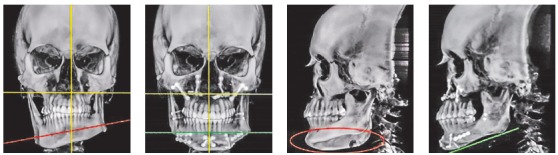
Tomographic scans before surgery and one year later.

**Figure 33:**
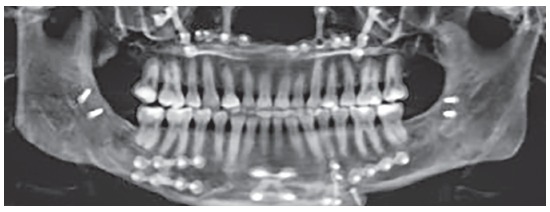
Panoramic radiograph after 1-year postoperative follow-up.

After postoperative follow-up (eight weeks), the patient was referred to postoperative orthodontic treatment for final alignment of teeth. One-year postoperative outcomes revealed inclination of mandibular and maxillary inferior borders was coinciding with the occlusal plane (Fig 31), thus showing facial symmetry and satisfactory occlusion. Imaging examination one year after surgery revealed the skeletal changes achieved (Figs 32 and 33).The literature claims virtual planning is a great tool for orthognathic surgery, yielding better final results than conventional planning.^6^ Particular attention should also be given to the use of 3D virtual platform for decision making during the orthodontic phase carried out in preparation for surgery. 

## CONCLUSION

Planning for orthognathic surgery does not comprise a single protocol; however, the literature and outcomes that have been published in the last few years highlight its importance as a tool that allows for accuracy and foreknowledge of results.

Cases reported in the present article suggest the 3D virtual platform should be used not only during surgical planning, but also a long time before that, aiding diagnosis of the deformity and allowing for assessment of feasible possibilities most likely to be recommended for patient’s orthodontic preparation. This shall ensure greater predictability.
